# The Effects of Malnutrition on Inpatient Outcomes in Patients With Gastroparesis: A Nationwide Analysis

**DOI:** 10.7759/cureus.47082

**Published:** 2023-10-15

**Authors:** Jay Patel, Kanwal Bains, Shivam Kalra, Ishandeep Singh, Isha Kohli, Dino Dukovic, Hunza Chaudhry, Aalam Sohal, Juliana Yang, Steven Tringali

**Affiliations:** 1 Internal Medicine, Digestive Disease and Surgical Institute, Cleveland Clinic, Cleveland, USA; 2 Internal Medicine, University of Arizona College of Medicine, Tucson, USA; 3 Internal Medicine, Trident Medical Center, North Charleston, USA; 4 Internal Medicine, Dayanand Medical College and Hospital, Ludhiana, IND; 5 Public Health Sciences, Icahn School of Medicine at Mount Sinai, New York, USA; 6 Internal Medicine, Ross University School of Medicine, Bridgetown, BRB; 7 Internal Medicine, University of California, Fresno, USA; 8 Hepatology, Liver Institute Northwest, Seattle, USA; 9 Gastroenterology and Hepatology, University of Texas Medical Branch, Galveston, USA

**Keywords:** national inpatient sample (nis), motility disorders, patient outcomes, malnutrition, gastroparesis

## Abstract

Introduction

Gastroparesis (GP) is a chronic debilitating gastric motility disorder defined as delayed emptying of the stomach content without mechanical obstruction. It can result in nutritional deficiencies, leading to poor overall outcomes. We assessed the impact of malnutrition on in-hospital outcomes in patients with gastroparesis.

Methods

Patients with a primary discharge diagnosis of GP between January 2016 and December 2019 were included in the National Inpatient Sample (NIS) database. Data on patient demographics, hospital characteristics, the Charlson Comorbidity Index (CCI), and the etiology of gastroparesis were collected. The association between malnutrition and outcomes, including mortality, deep vein thrombosis (DVT), pulmonary embolism (PE), sepsis, acute kidney injury (AKI), length of stay (LOS), and total hospitalization charges (THC), were analyzed using the multivariate regression model.

Results

A total of 182,580 patients with gastroparesis were included in the analysis. Patients with gastroparesis and malnutrition had a higher risk of mortality (adjusted odds ratio {aOR}, 3.29; p<0.001), sepsis (aOR, 0.43; p<0.001), DVT (aOR, 2.34; p<0.001), and PE (aOR, 2.68; p<0.001) compared to patients with gastroparesis without malnutrition. No significant difference was noted in the rates of AKI. Patients with malnutrition also had a prolonged LOS (2.96 days; p<0.001) and higher THC ($22,890; p<0.001) compared to patients without malnutrition.

Conclusion

Gastroparesis patients with malnutrition are at a greater risk of worse outcomes than those without malnutrition. The early identification of malnutrition in gastroparesis patients can predict morbidity and mortality and assist in risk stratification to enhance outcomes. Further studies are encouraged to identify factors associated with malnutrition in gastroparesis and the impact of interventions to prevent and treat malnutrition.

## Introduction

Gastroparesis (GP) is a chronic debilitating gastric motility disorder defined as the delayed emptying of stomach contents without any mechanical obstruction [1-3]. It presents with upper gastrointestinal symptoms such as nausea, vomiting, early satiety, postprandial pain, bloating, belching, and epigastric pain. Nausea and vomiting are considered the predominant symptoms of GP, while additional symptoms overlap with functional gastrointestinal disorders [4]. The exact pathophysiology is unknown, but the three most common etiologies are idiopathic, diabetic, and postsurgical [5]. Nutritional abnormalities are commonly seen in advanced stages of GP; therefore, the early screening and diagnosis of malnutrition remain critical [6,7].

The American Society for Parenteral and Enteral Nutrition (ASPEN) defines protein energy malnutrition (PEM) as two or more of the following: insufficient energy intake, weight loss, the loss of muscle mass and the loss of subcutaneous fat, localized or generalized fluid accumulation masking weight loss, and decreased functional status determined by handgrip strength [8]. The presence of malnutrition has been associated with worse outcomes in gastrointestinal conditions [9-11]. Malnutrition can affect the immune system, wound healing, and muscle mass, thus placing patients at higher risk for worse outcomes [12]. Previous studies have shown that malnutrition is associated with higher resource utilization and mortality [13].

We hypothesized that malnutrition would be associated with higher rates of death and adverse hospital events compared to those patients without malnutrition.

## Materials and methods

Data source

The National Inpatient Sample (NIS), maintained by the Healthcare Cost and Utilization Project (HCUP), is the largest database of inpatient hospital stays in the United States [14]. It contains information on 35 million weighted hospitalizations annually. Information regarding this data source has been discussed in previous studies [15,16]. Each hospitalization is de-identified and maintained in the NIS as a unique entry with one primary discharge diagnosis and up to 39 secondary diagnoses during that hospitalization, depending on the year of data collection. Each entry carries patient demographics, including age, sex, race, insurance status, primary and secondary procedures (up to 25), hospitalization outcome, total charges, and length of stay (LOS). An institutional review board (IRB) approval was not required as this study was conducted on publicly available de-identified data.

Study population

**Figure 1:**
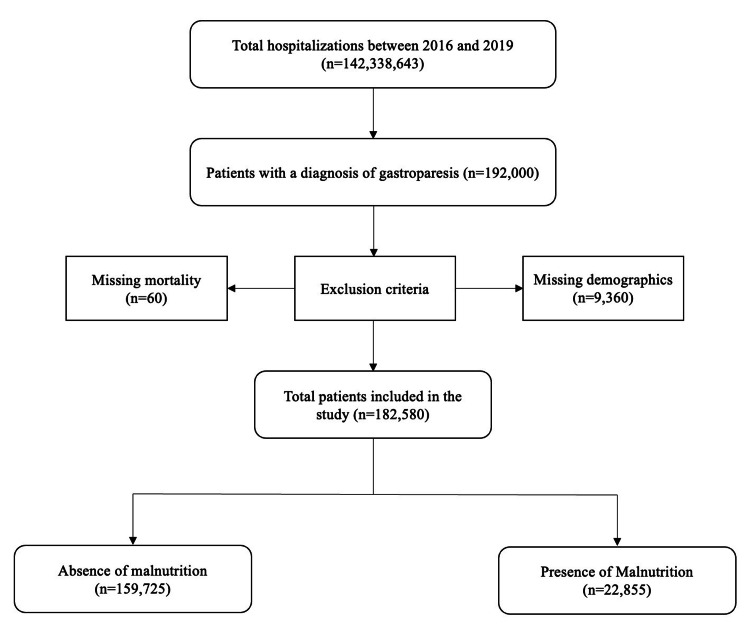
Flowchart of case selection for patients with gastroparesis.

We used the International Classification of Diseases, 10th Revision, Clinical Modification (ICD-10 CM) diagnosis codes to identify adult patients hospitalized with a primary diagnosis of gastroparesis between 2016 and 2019. We excluded cases with missing data on in-hospital mortality, gender, or demographic information. In total, 182,580 cases met the inclusion criteria. This information is presented in Figure 1.

Study outcomes and variables

The primary study outcome was inpatient mortality between malnourished and non-malnourished patients with GP. Secondary outcomes included rates of deep vein thrombosis (DVT), acute kidney injury (AKI), sepsis, and pulmonary embolism (PE). We also compared the mean LOS to total hospitalization charges (THC) between the two groups.

The nutritional status of the patient was the primary exposure variable, using ICD-10 codes for malnutrition (E40.x-E46.x, R63.4, and R64). Information was collected on age groups (divided into three groups: <44 years, 45-64 years, and >65 years), gender, race, primary insurance, median income, and hospital characteristics (region, bed size, and rural/urban location). Data were also collected on the etiology of GP based on the presence or absence of diabetes. The Elixhauser Comorbidity Index was used to assess the burden of comorbidities [17]. This is a well-validated index based on ICD 10-CM codes meant to be used in extensive administrative data to predict mortality and hospital resource use.

Statistical analysis

National estimates were generated using hospital discharge weights provided by the NIS. Chi-square and independent t-tests were used to compare categorical to continuous variables, respectively. Univariate logistic regression was performed to identify the association between malnutrition and categorical/continuous outcomes. Multivariate logistic regression was conducted while adjusting for patient demographics, hospital characteristics, Elixhauser comorbidities, and the etiology of GP for the variables that met the cutoff of p<0.1 on univariate analysis. The unadjusted and adjusted odds ratios (aOR) were reported with a 95% confidence interval (CI). A p-value of <0.05 was considered statistically significant. Stata 17.0 (StataCorp LLC, College Station, TX) was used for data analysis.

## Results

Patient demographics

Our study included 182,580 patients. Of the patients in the study population, 12.5% had malnutrition. Females accounted for 66.77% of the study population, and 70.16% had malnutrition. A higher proportion of malnourished patients were White (59.07%), followed by African American (23.15%) and Hispanic (12.89%) patients. Most of the malnourished patients had three or more comorbidities (46.08%). Further information is presented in Table 1.

**Table 1:** Patient demographics stratified by the presence of malnutrition.

Demographics	Absence of malnutrition, n (%)	Presence of malnutrition, n (%)	p-value
Age category			
18-44	72,120 (45.2)	9,270 (40.6)	<0.001
45-65	65,135 (40.8)	8,665 (37.9)	
>65	22,470 (14.1)	4,920 (21.5)	
Sex			<0.001
Male	53,860 (33.7)	6,860 (29.8)	
Female	105,865 (66.3)	16,035 (70.2)	
Race			<0.001
White	73,275 (45.9)	13,500 (59.1)	
African American	57,215 (35.8)	5,290 (23.2)	
Hispanic	21,605 (13.5)	2,945 (12.9)	
Asian/Pacific Islander	2,335 (1.5)	450 (2.0)	
Native American	935 (0.6)	95 (0.4)	
Other	4,360 (2.7)	575 (2.5)	
Insurance			<0.001
Medicare	66,950 (41.9)	10,040 (43.9)	
Medicaid	42,515 (26.6)	5,790 (25.3)	
Private	36,885 (23.1)	5,765 (25.2)	
Uninsured	9,590 (6.0)	800 (3.5)	
Income			<0.001
Lowest quartile	63,310 (39.6)	7,755 (33.9)	
Second quartile	41,805 (26.2)	6,240 (2.7)	
Third quartile	33,750 (21.1)	5,320 (23.3)	
Highest quartile	20,860 (13.1)	3,540 (15.5)	
Hospital region			<0.001
Northeast	24,805 (15.5)	3,505 (15.3)	
Midwest	27,800 (17.4)	5,075 (22.2)	
South	79,730 (49.9)	9,445 (41.3)	
West	27,390 (17.2)	4,830 (21.1)	
Hospital location			<0.001
Rural	11,060 (6.9)	1,050 (4.6)	
Urban	148,665 (93.1)	21,805 (95.4)	
Hospital teaching status			<0.001
Nonteaching hospitals	47,695 (29.9)	5,185 (22.7)	
Teaching hospitals	112,030 (70.1)	17,670 (77.3)	
Bed size of hospital			0.0005
Small	31,495 (19.7)	3,715 (16.3)	
Medium	50,080 (31.4)	6,840 (29.9)	
Large	78,150 (48.9)	12,300 (53.8)	
Elixhauser comorbidities			<0.001
0	15,510 (9.7)	4,400 (19.3)	
1	8,840 (5.5)	2,515 (11.0)	
2	53,820 (33.7)	6,095 (26.7)	
3 or more	81,555 (51.1)	9,845 (43.1)	

Etiology of gastroparesis

The majority of the patients had diabetic GP (76.22%), while the remaining had non-diabetic GP (23.78%). About 43.16% of the patients in the malnourished group had diabetic GP, while 56.84% of patients in the malnourished group had non-diabetic GP. Further information is presented in Table 2.

**Table 2:** Etiology of gastroparesis, stratified by the presence of malnutrition.

	Absence of malnutrition, N (%)	Presence of malnutrition, N (%)	p-value
Diabetic gastroparesis	126,165 (79.0)	9,865 (43.2)	<0.001
Non-diabetic gastroparesis	33,560 (21.0)	12,990 (56.8)	<0.001

Outcomes

Deaths

The total number of deaths in the study population was 435 (0.24%). The number of deaths in patients with malnutrition was 165 (0.72%) compared to 270 (0.17%) in those without malnutrition. A statistically significant higher risk of mortality was noted in patients with malnutrition than in those without (adjusted odds ratio {aOR}, 3.29; 95% confidence interval {CI}, 2.05-5.28; p<0.001). The results of the outcomes are presented in Table 3.

**Table 3:** Unadjusted and adjusted odds ratios/coefficients for categorical and continuous outcomes in patients with gastroparesis, stratified by the presence of malnutrition. AKI, acute kidney injury; DVT, deep vein thrombosis; PE, pulmonary embolism; LOS, length of stay

Categorical outcomes	Absence of malnutrition, N (%)	Presence of malnutrition, N (%)	p-value	Unadjusted odds ratio	p-value	Adjusted odds ratio	p-value
Death	270 (0.2)	165 (0.7)	<0.001	4.29 (2.76-6.66)	<0.001	3.29 (2.05-5.28)	<0.001
Sepsis	2,225 (1.4)	420 (1.8)	0.049	1.32 (1.00-1.75)	0.05	1.43 (1.08-1.90)	0.013
AKI	31,750 (19.9)	3,920 (17.2)	<0.001	0.83 (0.76-0.91)	<0.001	0.98 (0.89-1.07)	0.667
DVT	1,435 (0.9)	525 (2.3)	<0.001	2.59 (2.05-3.27)	<0.001	2.34 (1.85-2.96)	<0.001
PE	270 (0.2)	125 (0.6)	<0.001	3.24 (2.02-5.20)	<0.001	2.68 (1.63-4.41)	<0.001
Continuous outcomes				Unadjusted coefficient	p-value	Adjusted coefficient	p-value
LOS	3.9 (3.8-3.9)	7.0 (6.8-7.3)	<0.001	3.15 (2.88-3.42)	<0.001	2.96 (2.70-3.23)	<0.001
Total hospitalization charges	7.0 (6.8-7.3)	61,177.8 (58,440.4-63,915.2)	<0.001	24,946.61 (22,315.67-27,577.55)	<0.001	22,890.56 (20,399.18-25,381.94)	<0.001

Sepsis

The incidence of sepsis in the study population was 2,645 (1.45%). The incidence of sepsis in patients with malnutrition was 1.83% compared to 1.40% in patients without malnutrition. Compared to patients without malnutrition, patients with malnutrition had a statistically significant higher risk of developing sepsis (aOR, 1.43; 95% CI, 1.08-1.90; p=0.013).

Acute Kidney Injury (AKI)

The total number of patients who developed AKI in the study population was 35,670 (19.54%). There were 3,920 (17.15%) patients with malnutrition and 31,750 (19.88%) patients without malnutrition. Compared to patients without malnutrition, patients with malnutrition did not have a statistically significant higher risk of AKI (aOR, 0.98; 95% CI, 0.89-1.07; p=0.667).

Deep Vein Thrombosis (DVT)

The incidence of DVT in the study population was 1,960 (1.07%). It was 525 (2.30%) in malnourished patients and 1,435 (0.90%) in patients without malnutrition. Patients with malnutrition had a 2.34 higher risk of developing DVT than those without (95% CI, 1.85-2.96; p<0.001).

Pulmonary Embolism (PE)

The total number of patients who developed PE was 395 (0.22%). One hundred twenty-five (0.55%) patients with malnutrition and 270 (0.17%) patients without malnutrition developed PE. Compared to patients without malnutrition, malnourished patients had a 2.68 times higher risk of developing PE (95% CI, 1.63-4.41; p<0.01).

Length of Stay (LOS)

Patients with malnutrition had an average length of stay of 7.04 (±0.14) days, compared to 3.88 (±0.02) days for patients without malnutrition. Compared to patients without malnutrition, malnourished patients had a statistically significant higher LOS (adjusted coefficient, 2.96; 95% CI, 2.70-3.23; p<0.001).

Total Hospitalization Charges

The mean total hospitalization charges in patients with malnutrition were $61,177.79 compared to $36,231.17 in patients without malnutrition. Malnourished patients had statistically significantly higher total hospitalization charges than those without malnutrition (adjusted coefficient, $22,890.56; 95% CI, 20,399.18-25,381.94; p<0.001).

## Discussion

Our study is the first to evaluate the effect of malnutrition on hospital outcomes in patients with GP. Patients with malnutrition had a higher risk of mortality than those without malnutrition (aOR: 3.29). Various studies have found that malnutrition significantly predicts mortality in patients without a GP [18-20]. Malnutrition due to GP can result in vitamin and mineral deficiencies [21]. Patients with malnutrition are also at increased risk of iron deficiency. Vitamin and mineral deficiencies have been linked to mortality in hospitalized patients and patients undergoing major surgeries [22]. Malnutrition may also increase mortality through other mechanisms, such as hypoglycemia and hypothermia [23,24]. Patients with malnutrition who cannot tolerate oral feeding should receive alternative forms of enteral nutrition to improve outcomes [25]. It is critical to improve the malnutrition status of patients with GP.

Patients with malnutrition were also noted to be at an increased risk of thrombotic events such as DVT and PE. This is significant as gastroparesis patients at baseline have been shown to have increased thromboembolic risk as compared to controls [26]. Malnutrition in itself is a known risk factor for DVT and PE in patients with gastrointestinal conditions [27,28]. Studies have shown a statistically significant relationship between the risk of thrombosis and nutritional indices such as BMI, weight, and waist circumference [29]. The reasons for these associations are unclear; however, multiple hypotheses have been postulated. Dietary intake influences factors VIIc and VIIIc and the Von Willebrand factor, which are related to the risk of venous thromboembolism [30]. According to Folsom et al., venous thromboembolism is associated with low serum albumin [31]. Multiple other studies have reported conflicting results; however, these were small, cross-sectional or retrospective clinical studies [32-36]. Low albumin has been associated with higher fibrinogen and factor VIII levels, reflecting a hypercoagulable state [31]. Low serum albumin likely reflects poor general health, which itself predisposes to DVT. A deficiency of vitamin D can also increase the risk of DVT [37]. Furthermore, vitamin B12 and folate deficiency lead to the increased production of homocysteine, which has been involved in the thrombotic process [38,39]. Further studies investigating this association are necessary.

While previous studies have shown that malnutrition is associated with an increased risk of developing AKI, the analysis of this population did not reveal an associated increased risk of AKI [40]. This could be due to the under-recognition of AKI in malnourished patients [41]. Serum creatinine is reflective of somatic protein stores, and these levels may be decreased in patients with malnourishment due to decreased muscle mass. Our study also noted that malnutrition was associated with a higher risk of sepsis (aOR: 1.43). Previous studies have shown a stronger link between malnutrition and infection [42-45]. In addition, Katona and Katona-Apte explain that patients with infection have inadequate dietary intake and high energy requirement, which can further lead to lowered immunity. Other studies have also documented that patients with sepsis and malnutrition are associated with increased in-hospital mortality [43].

Patients with malnutrition were also noted to have a prolonged LOS and higher hospitalization resources. As previously described, malnutrition is associated with increased rates of sepsis, death, and thrombotic events. These patients had higher disease severity than those without malnutrition, which might have played a role in higher LOS and total hospitalization charges. Additionally, patients with malnutrition are at increased risk of disposition to nursing care facilities. In a study by Bell et al., more than 20% of nursing home residents had malnutrition [46]. Discharge planning may require coordination between physicians, social workers, and nursing homes, leading to prolonged length of stay [45].

This NIS-based study has several strengths and limitations. The strengths include the ability to assess a study population, which is nationally represented and derived based upon the largest publicly available inpatient database. Through this, we are able to produce regional and national statistics and estimates on patient outcomes, inpatient utilization, and healthcare costs, among others. The study was designed to assess various outcome measures comparing gastroparesis patients with and without malnutrition. This not only allows for the assessment of the effect of malnutrition on the measures and outcomes of gastroparesis patients but also provides an estimate of the degree of disease and burden on the healthcare system.

The NIS database does not contain information regarding the severity of the disease or the methods to establish the diagnosis. It lacks information on pharmaceutical therapies used during hospitalization. Since the NIS uses ICD-10 codes to identify patients, coding errors cannot be excluded. In addition, the degree of malnutrition was unable to be assessed, and overall numbers may be underreported. This database only includes present hospitalization data, and therefore, readmissions cannot be evaluated. The study did not differentiate between various etiologies of gastroparesis, such as diabetic versus non-diabetic. However, despite limitations, the large population sample size and the analysis of various outcomes provide a better assessment and understanding of the association between malnutrition and gastroparesis. The findings of this study should be validated in a prospective cohort study that captures more detailed clinical information about treatment and long-term mortality.

## Conclusions

This study expands on the importance of nutritional status in the outcomes of patients with GP. Our results reveal that malnutrition is common in patients with GP. A strong association between malnutrition and worse outcomes such as in-hospital mortality, sepsis, DVT, PE, and prolonged hospital stay was noted. Malnutrition assessment is a critical component in hospitalized patients. Systematic approaches should be considered in patients with GP, including nutritional screening, dietary recommendations, medical therapy, and options such as enteral or parenteral nutrition.
